# Erratum: Catalyzed Synthesis of Zinc Clays by Prebiotic Central Metabolites

**DOI:** 10.1038/s41598-017-04859-3

**Published:** 2017-08-02

**Authors:** Ruixin Zhou, Kaustuv Basu, Hyman Hartman, Christopher J. Matocha, S. Kelly Sears, Hojatollah Vali, Marcelo I. Guzman

**Affiliations:** 1 Department of Chemistry, University of Kentucky, Lexington, KY 40506 USA; 2 Facility for Electron Microscopy Research, McGill University, 3640 University Street, Montreal, Quebec, H3A 0C7 Canada; 3 Earth, Atmosphere, and Planetary Science Department, Massachusetts Institute of Technology, Cambridge, MA 02139 USA; 4 Department of Plant and Soil Sciences, University of Kentucky, Lexington, KY 40546 USA; 5 Department of Anatomy & Cell Biology, McGill University, 3640 University Street, Montreal, Quebec, H3A 0C7 Canada

*Scientific Reports* **7**:533; doi:10.1038/s41598-017-00558-1; Article published online 03 April 2017

In the original version of this Article, Hojatollah Vali was incorrectly affiliated to ‘Department of Chemistry, University of Kentucky, Lexington, KY, 40506, USA’ The correct affiliations for this author are listed below:

Facility for Electron Microscopy Research, McGill University, 3640 University Street, Montreal, Quebec, H3A 0C7, Canada.

Department of Anatomy & Cell Biology, McGill University, 3640 University Street, Montreal, Quebec﻿,﻿ H3A 0C7, Canada.

This error has now been corrected in the PDF and HTML versions of this Article.

