# Supplementary material for: Catalyzed Synthesis of Zinc Clays by Prebiotic Central Metabolites
Source: Sci Rep. 2017 Apr 3;7:533. doi: 10.1038/s41598-017-00558-1 (PMC5428702; doi:10.1038/s41598-017-00558-1)
Supplement: Supplementary file 5 — Supplementary Information [file 41598_2017_558_MOESM5_ESM.pdf]

Supplementary Information for:

Catalyzed Synthesis of Zinc Clays by Prebiotic Central Metabolites

Ruixin Zhou[a], Kaustuv Basu[b], Hyman Hartman[c], Christopher J. Matocha[d], S. Kelly Sears[b], Hojatollah Vali[b,e], and Marcelo I. Guzman*[,a]

*Corresponding Author: marcelo.guzman@uky.edu

[a]Department of Chemistry, University of Kentucky, Lexington, KY, 40506, USA; [b]Facility for Electron Microscopy Research, McGill University, 3640 University Street, Montreal, Quebec H3A 0C7, Canada; [c]Earth, Atmosphere, and Planetary Science Department, Massachusetts Institute of Technology, Cambridge, MA 02139, USA; [d]Department of Plant and Soil Sciences, University of Kentucky, Lexington, KY, 40546, USA; [e]Department of Anatomy & Cell Biology, 3640 University Street, Montreal H3A 0C7, Canada

Number of *SI* pages: 7

Number of *SI* figures: 9

Number of *SI* Videos: 4

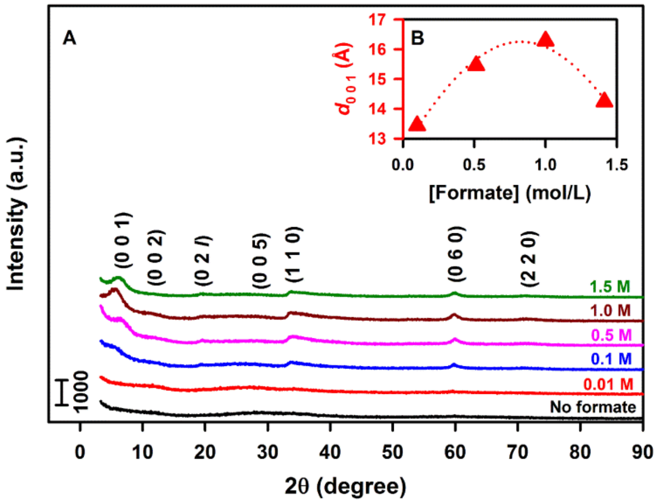

Figure S1. (A) Powder XRD diffractograms of sauconite synthesized at pH_0 6.3 at 90 °C during 20 h under the variable [formate] listed above each trace. Subscript numbers, e.g., (0 0 1), indicate basal reflections of the identified phases. (B) The layer to layer distance for 2:1 sauconite ($d_{0\,0\,1}$, red solid triangle) for traces in (A) vs [formate].

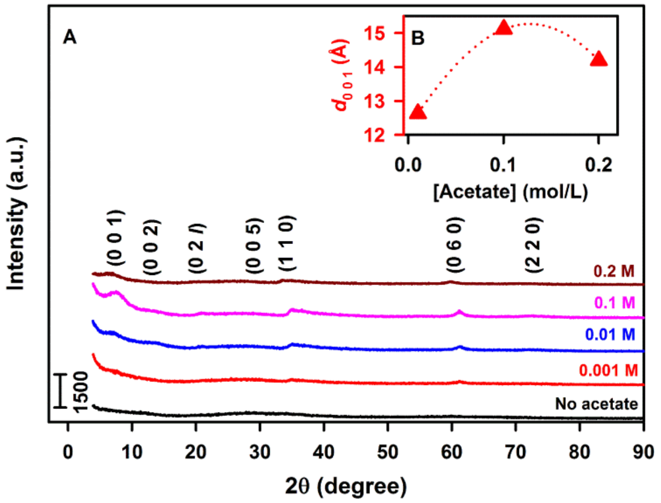

Figure S2. (A) Powder XRD diffractograms of sauconite synthesized at pH_0 6.3 at 90 °C during 20 h under the variable [acetate] listed above each trace. Subscript numbers, e.g., (0 0 1), indicate basal reflections of the identified phases. (B) The layer to layer distance for 2:1 sauconite ($d_{0\,0\,1}$) for traces in (A) vs [acetate].

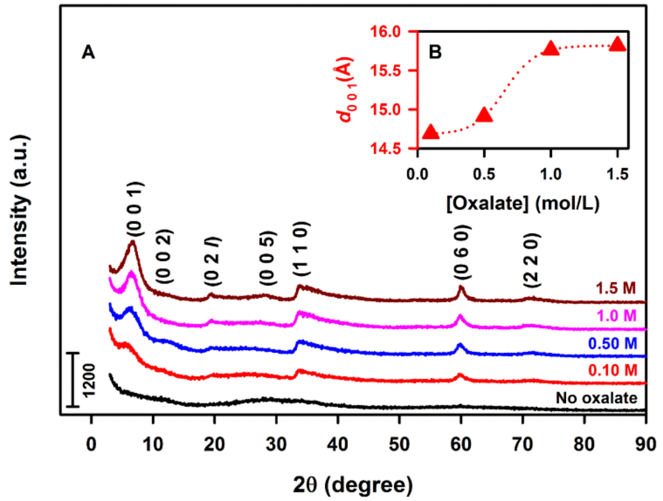

Figure S3. (A) Powder XRD diffractograms of sauconite synthesized at pH_0 6.3 at 90 °C during 20 h under the variable [oxalate] listed above each trace. Subscript numbers, e.g., (0 0 1), indicate basal reflections of the identified phases. (B) The layer to layer distance for 2:1 sauconite ($d_{0\,0\,1}$) for traces in (A) vs [oxalate].

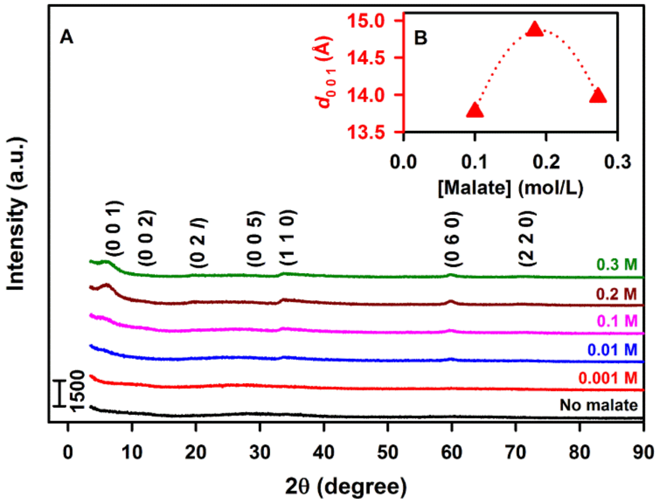

Figure S4. (A) Powder XRD diffractograms of sauconite synthesized at pH_0 6.3 at 90 °C during 20 h under the variable [malate] listed above each trace. Subscript numbers, e.g., (0 0 1), indicate basal reflections of the identified phases. (B) The layer to layer distance for 2:1 sauconite ($d_{0\,0\,1}$) for traces in (A) vs [malate].

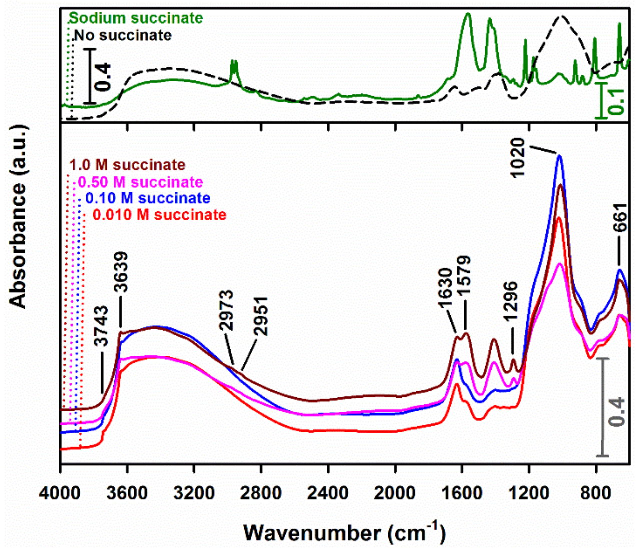

Figure S5. DRIFT spectra of sauconite synthesized under variable [succinate] for the same conditions of Figure 1 (20 h at pH_0 6.3 and 90 °C) compared to sodium succinate and the starting gel.

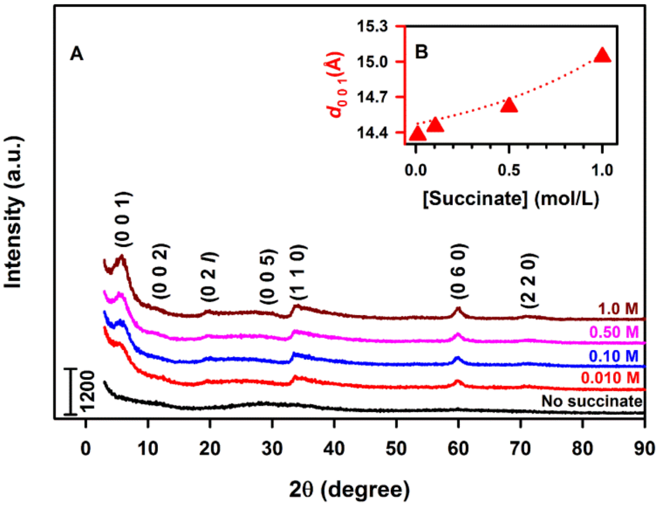

Figure S6. (A) Powder XRD diffractograms of sauconite synthesized at pH_0 6.3 at 90 °C during 20 h dried under room temperature instead of in the oven as in the other Figures. The variable [succinate] is listed above each trace. (B) The layer to layer distance for 2:1 sauconite ($d_{0\,0\,1}$) vs [succinate].

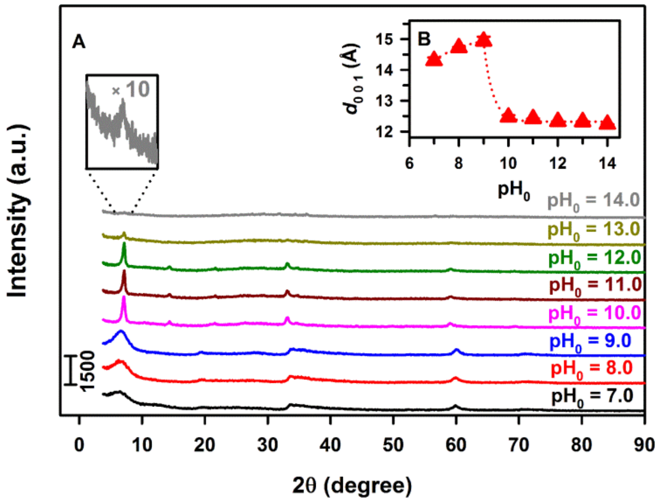

Figure S7. (A) Powder XRD diffractograms of sauconite synthesized with 1.0 M succinate at 90 °C during 20 h under the variable pH_0 values indicated over the traces. (B) The layer to layer distance for 2:1 sauconite, $d_{0\,0\,1}$ from 2θ = 5.82° is (A) vs pH_0.

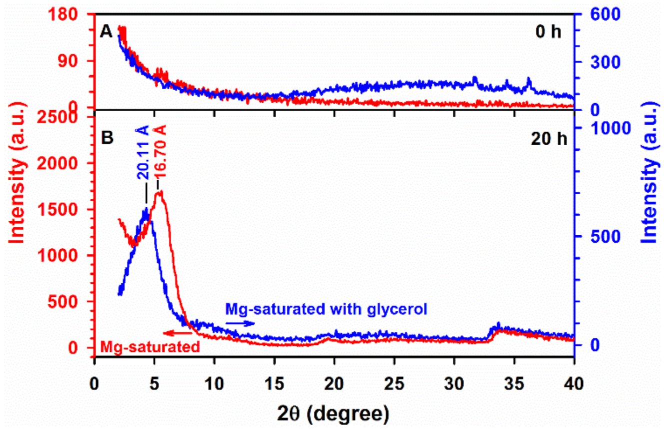

Figure S8. XRD patterns of (red) Mg-saturated and (blue) Mg-saturated with glycerol of synthesized sauconite with 1.0 M succinate at pH_0 9.0 and 90 °C for timepoints at (A) 0 and (B) 20 h.

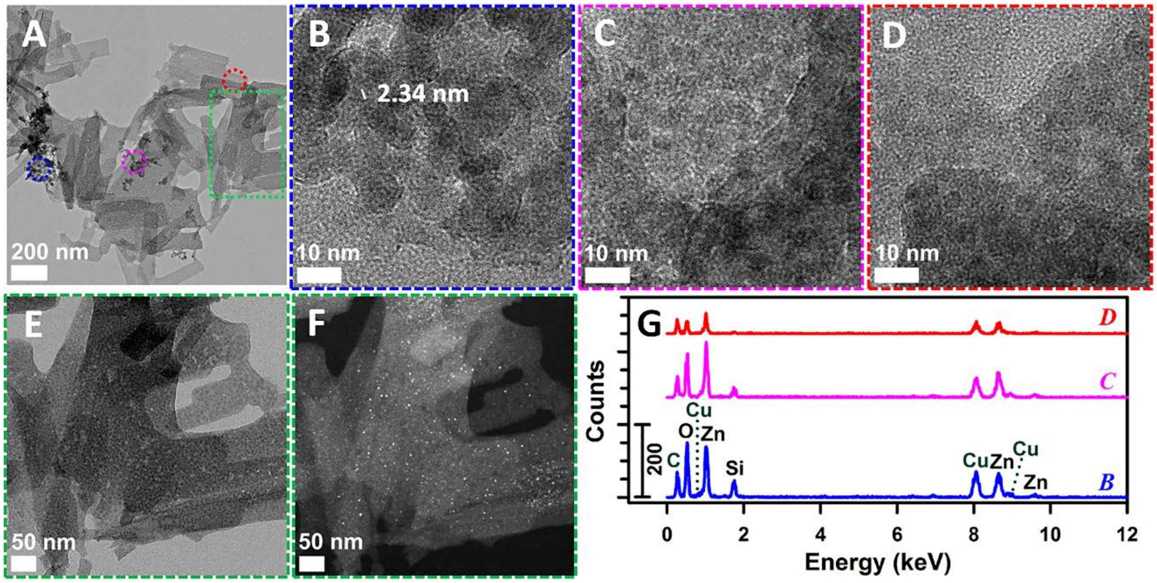

Figure S9. Transmission electron micrographs (TEM) and energy dispersive X-ray spectra (EDS) of initial gel with 1.0 M succinate and pH_0 9.0 before starting reflux showing morphologies in different parts. (A) Large region showing the inhomogeneity of the gel with fours demarked sections displayed in close-ups: (B) blue circle for thick-agglomerated microspheres; (C) pink circle for thin-agglomerated microspheres; (D) red circle for a more translucent region without microspheres; and (E and F) green square for the bright and dark field modes of a translucent region contrasting the presence of nanoscopic nuclei in the bulk structure but not on the surface. (G) EDS for the close-ups in panels A, B, and C.

Video M1. Different rotational views of the sample in Figure 7 after 20 h of synthesis.

Video M2. Different rotational views of the sample in Figure 7 after 20 h of synthesis.

Video M3. Tomograms of the sample displayed in Figure 7 after 20 h of synthesis.

Video M4. Tomograms of the sample displayed in Figure 5 after 20 h of synthesis.
